# First-line treatment for chronic myeloid leukemia: dasatinib, nilotinib, or imatinib

**DOI:** 10.1186/1756-8722-3-47

**Published:** 2010-11-26

**Authors:** Guoqing Wei, Shamudheen Rafiyath, Delong Liu

**Affiliations:** 1 Bone Marrow Transplantation Center, the First Affiliated Hospital, Zhejiang University School of Medicine, Hangzhou 310003, PR China; 2 Division of Hematology and Oncology, New York Medical College and Westchester Medical Center, Valhalla, NY 10595, USA

## Abstract

Imatinib, a tyrosine kinase inhibitor (TKI) of BCR-ABL, was the standard first-line therapy for chronic myeloid leukemia (CML) for almost 10 years. Dasatinib and nilotinib, two newer drugs with higher potency than imatinib against BCR-ABL and activity against most imatinib-resistant BCR-ABL mutations, have each shown superior efficacy compared with imatinib for first-line treatment of chronic-phase CML in randomized phase 3 trials. With 14 months follow-up time, available data suggest no obvious differences in efficacy between dasatinib and nilotinib. Compared with imatinib, dasatinib is associated with higher rates of pleural effusion and thrombocytopenia, but lower rates of edema, gastrointestinal AEs, musculoskeletal AEs, and rash. Nilotinib is associated with higher rates of dermatologic toxicity, headache, and biochemical abnormalities associated with hepatic and pancreatic toxicity compared with imatinib, but lower rates of edema, gastrointestinal AEs, muscle spasm, and neutropenia. Several studies have shown that poor adherence to imatinib detrimentally affects responses and should be considered in patients with a suboptimal response. The different dosing requirements of dasatinib (once daily with or without food) and nilotinib (twice daily with fasting) may be an additional factor in selecting frontline agents. This review compares and contrasts the three FDA approved first line TKI agents.

## Introduction

Imatinib, which inhibits the tyrosine kinase activity of BCR-ABL, was introduced as a first-line treatment for chronic myeloid leukemia (CML) almost 10 years ago and radically improved the outcome of patients with CML. Imatinib has been the standard therapy for CML due to its remarkable activity and mild toxicity. In the IRIS study (International randomized study of interferon vs STI571) of first-line treatment with imatinib or interferon and cytarabine in patients with newly diagnosed chronic phase (CP)-CML, patients in the imatinib arm had an 8-year overall survival rate of 85% and freedom from progression to advanced disease was 92% [1]. Imatinib was also generally well tolerated during long-term treatment.

Despite the responses observed with imatinib, a proportion of patients develops resistance to imatinib or cannot tolerate its side effects. This led to the development of newer tyrosine kinase inhibitors (TKIs) of BCR-ABL, including dasatinib, nilotinib, and bosutinib, that were initially tested in clinical studies of patients with prior imatinib therapy [2-5]. Dasatinib, nilotinib and bosutinib, respectively, have 325-fold, 20-30-fold, and 30-fold increased potency over imatinib against BCR-ABL kinase *in vitro *[6-9]. Nilotinib has a similar chemical structure to imatinib but has an improved topographical fit in the ABL kinase pocket [6,7,9]. Dasatinib has a completely different chemical structure to imatinib and, unlike imatinib and nilotinib, binds BCR-ABL in the active conformation [10,11]. Bosutinib binds to an intermediate form of BCR-ABL [8]. All three TKIs have activity against most of the mutated forms of BCR-ABL kinase that have been associated with clinical resistance to imatinib [6,9]. Dasatinib 100 mg once daily (QD) and nilotinib 400 mg twice daily (BID) have been approved in the US and Europe as treatments for patients with CML who are resistant or intolerant to imatinib (dasatinib for all phases of CML, nilotinib for CP and accelerated phase [AP]). Dasatinib 100 mg QD and nilotinib 300 mg BID were recently approved in the US for patients with newly diagnosed CP-CML. Bosutinib is still undergoing clinical trials.

Clinical trials assessing the newer TKIs (dasatinib, nilotinib, and bosutinib) as first-line therapies in newly diagnosed CP-CML are ongoing and results from trials of dasatinib and nilotinib have recently been reported. For dasatinib, published clinical trials in newly diagnosed CP-CML comprise: (i) DASISION (Dasatinib versus imatinib study in treatment-naive CML patients), an international, multicenter, randomized phase 3 trial of dasatinib 100 mg QD vs imatinib 400 mg QD (n = 519) [12]; and (ii) a single-arm phase 2 trial of dasatinib 100 mg QD or 50 mg BID performed by M D Anderson Cancer Center (MDACC), Houston, TX (n = 62) [13]. For nilotinib, published clinical trials in newly diagnosed CP-CML comprise: (i) ENESTnd (Evaluating nilotinib efficacy and safety in clinical trials - newly diagnosed patients), an international, multicenter, randomized phase 3 trial of nilotinib 300 mg BID vs nilotinib 400 mg BID vs imatinib 400 mg QD (n = 846) [14]; (ii) a single-arm phase 2 trial of nilotinib 400 mg BID performed by MDACC (n = 61) [15]; and (iii) a second single-arm phase 2 trial of nilotinib 400 mg BID performed by the Italian GIMEMA (Gruppo Italiano malattie e matologiche dell'adulto) group (n = 73) [4]. No data have been published from an international, multicenter, randomized trial of bosutinib vs imatinib (NCT00574873).

In this review, recent data for first-line treatment with dasatinib or nilotinib will be discussed, with a specific focus on safety and tolerability.

## Efficacy of dasatinib and nilotinib compared with imatinib in the first-line setting

In randomized trials, both dasatinib and nilotinib have shown superior efficacy compared with imatinib as first-line treatment for patients with CP-CML (Tables 1 and 2).

**Table 1:** Rates of complete cytogenetic response (CCyR) and major molecular response (MMR) to imatinib and dasatinib in the DASISION trial.

	% of patients	
	**Imatinib** **400 mg QD**	**Dasatinib** **100 mg QD**

CCyR		

3 months	31	54

6 months	59	73

9 months	67	78

12 months	72	83

MMR		

3 months	0.4	8

6 months	8	27

9 months	18	39

12 months	28	46

Progression to AP/BP	3.5	1.9

AP: accelerated phase; BP: blast phase; QD: once daily.

**Table 2:** Rates of complete cytogenetic response (CCyR) and major molecular response (MMR) to imatinib and nilotinib in the ENESTnd trial.

	% of patients		
	**Imatinib** **400 mg QD**	**Nilotinib** **300 mg BID**	**Nilotinib** **400 mg BID**

CCyR			

6 months	45	67	63

12 months	65	80	78

MMR			

3 months	1	9	5

6 months	12	33	30

9 months	18	43	38

12 months	22	44	43

Progression to AP/BP	4	< 1	< 1

AP: accelerated phase; BP: blast phase; QD: once daily.

In the DASISION trial, responses were more frequent with dasatinib vs imatinib treatment, including higher 12-month rates of complete cytogenetic response (CCyR; 83% vs 72%; P = 0.001) and major molecular response (MMR; 46% vs 28%; P < 0.0001). Dasatinib also showed superiority over imatinib in the primary trial endpoint, the rate of confirmed CCyR (CCyR detected in two consecutive assessments), with 12-month rates of 77% vs 66%, respectively (P = 0.007). CCyR and MMR both occurred faster with dasatinib compared with imatinib. After a median 14 months of treatment, 1.9% of patients had progressed to AP/blast phase (BP) with dasatinib compared with 3.5% with imatinib. No patient in whom a MMR was achieved progressed to AP/BP [12]. In the ENESTnd trial, the primary endpoint was the rate of MMR at 12 months, and both nilotinib arms (300 mg and 400 mg) had significantly higher rates compared with the imatinib arm (43-44% vs 22%; P < 0.001). Rates of CCyR achieved by 12 months were also significantly higher for nilotinib vs imatinib (78-80% vs 65%; P < 0.001), and CCyR and MMR occurred faster in the nilotinib arms. After a median 14 months of treatment, fewer nilotinib-treated patients had progressed to AP/BP phase compared with imatinib-treated patients (< 1% vs 4%; P ≤ 0.01 in an analysis of time to progression). Similar to DASISION, no patient who had a MMR had progression to AP/BP [14]. Five-year follow-up is planned in both trials. Because available data suggest that both dasatinib and nilotinib have broadly similar efficacy in terms of their superiority over imatinib, it is likely that safety and tolerability considerations for these agents will become increasingly important when selecting first-line treatment for CML.

## The importance of adherence

Across various chronic diseases requiring long-term treatment, poor adherence is associated with worse outcomes [16]. Similarly, recent studies have shown that lack of adherence to imatinib treatment results in significantly lower response rates in patients with CP-CML. In a prospective observational study (Adherence assessment with Glivec: indicators and outcomes; ADAGIO), adherence to imatinib treatment was analyzed in 169 patients with CML during a 90-day period and correlated with overall responses to treatment. Only 14% of patients were found to be perfectly adherent based on pill counts (100% of imatinib taken), with 71% of patients taking less imatinib than prescribed and 15% taking more imatinib than prescribed. Importantly, worse adherence was associated with worse treatment responses; patients who had a suboptimal response to imatinib had significant higher mean percentage of imatinib not taken than those with an optimal response (23% vs 7%; P = 0.005). Similarly, patients who failed to achieve a CCyR on imatinib had a higher mean percentage of pills not taken than patients who achieved a CCyR (24% vs 9%; P = 0.012) [17]. In another prospective observational study performed at a single institution, 87 patients with CP-CML who had achieved a CCyR on imatinib were monitored for adherence for 90 days using a microelectronic monitoring device. The adherence rate was ≤ 90% in 26% and ≤ 80% in 14%. There was a strong correlation between adherence to imatinib and probabilities of MMR and CMR; patients with ≤ 90% adherence had a lower 6-year rate of MMR than patients with > 90% adherence (14% vs 94%; P = 0.002), no patient with ≤ 90% adherence achieved a CMR, and no patient with ≤ 80% adherence achieved a MMR. Significantly worse adherence rates were found in patients with various adverse events (AEs), including asthenia, nausea, muscle cramps, and bone or joint pains, and also in patients who took imatinib independently of meals. Patients who had their imatinib dose increased had significantly worse adherence than patients who remained on imatinib 400 mg QD (86% vs 99%; P = 0.021) [18]. In a retrospective analysis of imatinib treatment in clinical practice using US administrative claims data, adherence to imatinib in 267 patients was calculated using the medication possession rate (MPR), ie, the total days supply of imatinib in a 1-year period divided by 365. Overall, the mean MPR was 78% and 31% of patients had a treatment interruption of at least 30 consecutive days. Among the study population, nonadherence was higher in patients with higher numbers of concomitant medications, women, patients with more complex disease, and patients with a higher starting dose of imatinib (≥ 600 mg/d). Although the reasons for worse adherence in women were not examined, the authors suggested that women may be more concerned than men with AEs characteristic of imatinib treatment, such as rash, edema, and weight gain [19].

The importance of adherence to imatinib in response to treatment is further illustrated by the results of a phase 3 randomized trial of imatinib 400 mg QD vs 800 mg/d (400 mg BID) in patients with newly diagnosed CP-CML (Tyrosine kinase inhibitor optimization and selectivity [TOPS]). Rates of MMR and CCyR at 12 months were similar between the two arms. However, treatment responses in patients from the 800 mg/d arm correlated with the dose of imatinib that could be tolerated, with higher MMR rates achieved in patients with an average dose intensity of 600 mg/d or higher (62-63%) compared with 400-599 mg/d (38%) or < 400 mg/d (21%). In the 400 vs 800 mg arms, 18% vs 61% of patients had a dose reduction, 52% vs 73% reported at least one day with zero dose, 38% vs 67% had dose interruption lasting longer than 5 days, and 16% vs 20% discontinued treatment. The main reason for dose reduction in the 800 mg/d arm, but not the 400 mg/d arm, was AEs or laboratory abnormalities. These data suggest that the higher number of days off medication (ie, lower adherence) in the high-dose imatinib arm counteracted any positive effect of higher dosing [20].

Nonadherence is a possible cause for reduced response to imatinib and should be considered in patients with suboptimal response to imatinib [17]. The AE profiles and tolerability of newer treatments are therefore important considerations for clinical practice in the first-line setting in terms of both efficacy and safety.

## Safety and tolerability of dasatinib and nilotinib compared with imatinib in the first-line setting

Although dasatinib and nilotinib have been available for use in therapy of CML in the second-line settings for several years, new studies have provided the first direct comparison with imatinib in the first-line setting. In general, imatinib, dasatinib, and nilotinib are associated with broadly similar types of AEs, although the relative occurrence of different AEs varies between agents and some AEs are specific to one drug (Tables 3 and 4). For best management of CML patients receiving TKI therapy, knowledge of potential toxicities, how to avoid them, how to deal with them should they arise, and how they may affect response and outcome, are important factors. In general, BCR-ABL inhibitors are well tolerated and result in a limited number of higher-grade toxicities (grades 3-4). Experience with imatinib in the IRIS trial and with dasatinib and nilotinib in the second-line setting suggest that AEs tend to occur early during the course of treatment and late-onset toxicity is uncommon [21-23]. Longer-term follow-up is needed to confirm that the same is true for dasatinib and nilotinib during first-line treatment. In general, most AEs occurring during BCR-ABL inhibitor therapy can be managed with dose interruption and reduction and/or supportive care.

**Table 3:** Drug-related nonhematologic adverse events, that occurred in ≥ 10% of patients in any treatment arm, hematologic adverse events, and biochemical abnormalities, during the DASISION trial.

	All grade (grade 3-4), % of patients	
**Adverse event**	**Imatinib** **400 mg QD**	**Dasatinib** **100 mg QD**

Nonhematologic		

Nausea	20 (0)	8 (0)

Diarrhea	17 (1)	17 (< 1)

Vomiting	10 (0)	5 (0)

Rash	17 (1)	11 (0)

Headache	10 (0)	12 (0)

Fatigue	10 (0)	8 (< 1)

Musculoskeletal pain	14 (< 1)	11 (0)

Muscle inflammation	17 (< 1)	4 (0)

Fluid retention	42 (1)	19 (1)

Superficial edema	36 (< 1)	9 (0)

Pleural effusion	0 (0)	10 (0)

Other	8 (< 1)	5 (1)

Hematologic		

Neutropenia	58 (20)	65 (21)

Thrombocytopenia	62 (10)	70 (19)

Anemia	84 (7)	90 (10)

Biochemical abnormalities		

Elevated AST	NL (1)	NL (< 1)

Elevated ALT	NL (1)	NL (< 1)

Elevated bilirubin	NL (0)	NL (1)

Elevated lipase	NL (0)	NL (0)

Hyperglycemia	NL (0)	NL (0)

Elevated amylase	NL (0)	NL (0)

Decreased phosphorus	NL (21)	NL (4)

ALT: alanine aminotransferase; AST: aspartate aminotransferase; NL: not listed; QD: once daily.

**Table 4:** Drug-related nonhematologic adverse events, that occurred in ≥ 10% of patients in any treatment arm, hematologic adverse events, and biochemical abnormalities, during the ENESTnd trial.

	All grade (grade 3-4), % of patients		
**Adverse event**	**Imatinib** **400 mg QD**	**Nilotinib** **300 mg BID**	**Nilotinib** **400 mg BID**

Nonhematologic			

Nausea	31 (0)	11 (< 1)	19 (1)

Diarrhea	21 (1)	8 (1)	6 (0)

Vomiting	14 (0)	5 (0)	9 (1)

Rash	11 (1)	31 (< 1)	36 (3)

Pruritus	5 (0)	15 (< 1)	13 (< 1)

Alopecia	4 (0)	8 (0)	13 (0)

Headache	8 (0)	14 (1)	21 (1)

Fatigue	8 (< 1)	11 (0)	9 (1)

Muscle spasm	24 (1)	7 (0)	6 (1)

Myalgia	10 (0)	10 (< 1)	10 (0)

Peripheral edema	14 (0)	5 (0)	5 (0)

Eyelid edema	13 (< 1)	1 (0)	2 (< 1)

Periorbital edema	12 (0)	< 1 (0)	1 (0)

Hematologic			

Neutropenia	68 (20)	43 (12)	38 (10)

Thrombocytopenia	56 (9)	48 (10)	49 (12)

Anemia	47 (5)	38 (3)	38 (3)

Biochemical abnormalities			

Elevated AST	23 (1)	40 (1)	48 (3)

Elevated ALT	20 (2)	66 (4)	73 (9)

Elevated bilirubin	10 (< 1)	53 (4)	62 (8)

Elevated lipase	11 (3)	24 (6)	29 (6)

Hyperglycemia	20 (0)	36 (6)	41 (4)

Elevated creatinine	13 (< 1)	5 (0)	5 (0)

Elevated amylase	12 (1)	15 (< 1)	18 (1)

Elevated alkaline phosphatase	33 (< 1)	21 (0)	27 (0)

Decreased phosphorus	45 (8)	32 (5)	34 (5)

ALT: alanine aminotransferase; AST: aspartate aminotransferase; BID: twice daily; QD: once daily.

## Cytopenias

Cytopenias such as neutropenia, thrombocytopenia, and anemia are the most common grade 3-4 AEs observed in patients receiving imatinib, dasatinib, or nilotinib. In the DASISION trial, grade 3-4 cytopenia with dasatinib vs imatinib included similar rates of neutropenia (20% vs 21%) and anemia (10% vs 7%), whereas thrombocytopenia was more common with dasatinib than with imatinib (19% vs 10%) [12]. Few patients discontinued treatment due to cytopenia (1.5% with dasatinib and 1.2% with imatinib) [12]. In the MDACC study of dasatinib, grade 3-4 neutropenia, thrombocytopenia, and anemia occurred in 21%, 10%, and 6% of patients, respectively [13]. In the ENESTnd trial, grade 3-4 neutropenia was less common in the nilotinib 300 or 400 mg BID arms (12% and 10%, respectively) compared with the imatinib arm (20%), whereas grade 3-4 thrombocytopenia (10% vs 12% vs 9%) and anemia (3% vs 3% vs 5%) were similar between treatment arms [14]. In the MDACC study of nilotinib, grade 3-4 neutropenia, thrombocytopenia, and anemia occurred in 12%, 11%, and 5% of patients, respectively [15], whereas low rates (4%, 2%, and 0%) were reported in the GIMEMA study [4].

## Dermatologic toxicity

Rash was one of the most common nonhematologic AEs [24,25]. In the IRIS study, rash occurred in 34%, although grade 3-4 rash was infrequent (2%). Pruritus (7%) and alopecia (4%) were also noted in smaller numbers of patients [25]. In the DASISION trial, first-line dasatinib treatment resulted in fewer cases of rash compared with imatinib treatment (11% vs 17%), with grade 3-4 rash occurring in 0% vs 1%, respectively. No rates were provided for pruritis or alopecia, suggesting that the frequencies were < 10% in both arms [12]. In the MDACC study, 58% of patients experienced "skin toxicity" (grouped term) with dasatinib, which was grade 3-4 in 2%. In addition, 8% experienced pruritus of which 2% was grade 3-4 [13]. Dermatologic toxicity seems to be more common with nilotinib than imatinib. In the ENESTnd trial, rash occurred in 31% taking nilotinib 300 mg BID, 36% taking nilotinib 400 mg BID, and 11% taking imatinib (grade 3-4 in < 1% vs 3% vs 1%, respectively). Pruritus was also more common in both nilotinib arms (15% with 300 mg BID and 13% with 400 mg BID) compared with imatinib (5%), as was alopecia (8% with nilotinib 300 mg BID, 13% with nilotinib 400 mg BID, and 4% with imatinib) [14]. In single-arm trials of first-line nilotinib 400 mg BID, rash occurred in 49% (2% grade 3-4) of patients in the MDACC trial [15] and in 42% (5% grade 3) in the GIMEMA trial [4]. Pruritus also occurred in 21% of patients in the GIMEMA trial (4% grade 3).

## Gastrointestinal symptoms

Nausea, diarrhea, and vomiting are common in patients receiving BCR-ABL inhibitor therapy, although recent data indicate that gastrointestinal (GI) disturbances occur less often in patients receiving dasatinib or nilotinib compared with those receiving imatinib. In the DASISION trial, nausea (8% v 20%) and vomiting (5% vs 10%) both occurred less frequently with dasatinib compared with imatinib, whereas rates of diarrhea were similar (17% in both arms). Grade 3-4 diarrhea was reported in < 1-1%, and no patients in either arm experienced grade 3-4 nausea or vomiting [12]. In the MDACC trial of dasatinib, higher rates of GI AEs were reported, including diarrhea in 53% (2% grade 3-4), nausea in 45% (0% grade 3-4), and vomiting in 21% (0% grade 3-4) [13]. In the ENESTnd trial, rates of GI AEs were lower with nilotinib 300 mg and 400 mg vs imatinib, including nausea (11% vs 19% vs 31%), diarrhea (8% vs 6% vs 21%), and vomiting (5% vs 9% vs 14%), of which 0-1% were grade 3-4 cases in all arms [14]. In the MDACC study of first-line nilotinib, nausea and diarrhea were reported in 38% and 21% of patients, respectively, (no grade 3-4), and diarrhea occurred in 7% (2% grade 3-4) [15]. In the GIMEMA study, 11% of patients experienced nausea/vomiting (1% grade 3-4) and 7% had diarrhea (2% grade 3) [4].

## Edema

Fluid retention is common with imatinib, as shown by 56% of patients receiving imatinib in the IRIS trial experiencing superficial edema and 13% having weight gain [25]. First-line dasatinib and nilotinib treatment are associated with lower rates of edema. In the DASISION, superficial edema (grouped term) was much less frequent with dasatinib (9%) compared with imatinib (36%), and rates of grade 3-4 superficial edema were low (0% vs < 1%, respectively) [12]. In the MDACC study of dasatinib, edema was reported in 32% of patients (no grade 3-4) [13]. In the ENESTnd trial, different types of edema were reported separately. In the nilotinib 300 mg BID, nilotinib 400 mg BID, and imatinib arms, peripheral edema occurred in 5% vs 5% vs 14%, eyelid edema occurred in 1% vs 2% vs 13%, and periorbital edema occurred in < 1% vs 1% vs 12% [14]. In the GIMEMA trial, peripheral edema was reported in 4% of patients receiving nilotinib and all cases were grade 1-2 [4]. Data for edema were not reported in the MDACC study of nilotinib [15].

## Pleural effusion

Pleural effusion is rare with nilotinib and imatinib but is a more prominent side effect of dasatinib treatment [26,27]. In the DASISION trial, 10% of patients in the dasatinib arm had a pleural effusion whereas no patient receiving imatinib reported this AE. Dasatinib-associated pleural effusion was grade 1 in 2% and grade 2 in 8% of patients, with no pleural effusion grade 3 or above. The occurrence of pleural effusion did not affect the efficacy of dasatinib, as shown by CCyR being achieved in 24/26 patients (92%) who had a pleural effusion. In the DASISION trial, pleural effusion was managed using dose adjustments and/or medical intervention, including dose interruption in 19 patients, diuretics in 12 patients, dose reduction in eight patients, corticosteroids in seven patients, and therapeutic thoracentesis in one patient. Discontinuation due to pleural effusion occurred in three patients (1% of the dasatinib arm) [12]. In the MDACC study of first-line dasatinib, the rate of pleural effusion (13%) was similar to DASISION, and one case of grade 3/4 pleural effusion was reported. Pleural effusion occurred less frequently in patients who received dasatinib 100 mg QD (6%) compared with 50 mg BID (19%), and two patients (3%) discontinued treatment due to pleural effusion [13]. In the ENESTnd study, pleural effusion occurred in a small number (< 1%) of nilotinib-treated patients [28] and was not reported in the single-arm studies of nilotinib.

## Cardiac toxicity

In 2006, a report was published describing ten individuals who developed severe congestive heart failure (CHF) on imatinib treatment. Based on laboratory studies, the authors suggested that this effect could occur as a result of inhibition of physiologic ABL activity in cardiac tissue [29]. Subsequent retrospective analyses estimated that the frequency of CHF or left ventricular dysfunction during imatinib therapy for CML was 0.5-1.1% [30-32].

In TKI studies, instances of QT prolongation were reported [33-37]. In particular, in studies of nilotinib in patients with imatinib resistance or intolerance, sudden death was reported in 0.6% of patients, with a similar rate of occurrence in an expanded-access program. The timing of sudden death relative to initiation of nilotinib suggested that ventricular repolarization abnormalities may have contributed to their occurrence [34]. In recent TKI trials, patients with significant cardiac disease were excluded from participating.

In randomized trials of nilotinib or dasatinib vs imatinib, close monitoring for QT prolongation and changes in left ventricular ejection fraction was performed. During nilotinib or imatinib treatment in the ENESTnd study, no patient had a QTc interval of > 500 msec and no decrease from the baseline in the mean left ventricular ejection fraction was observed at any time. Eleven patients across all three study arms had an ischemic heart disease event, although no further details were provided regarding relative frequency between arms [14]. In the MDACC study of front-line nilotinib, there were two instances of hypertension and one instance of QTc prolongation (all classed as grade 1-2) [15]. In the GIMEMA study of nilotinib, 584 electrocardiograms from 73 patients were reviewed. In addition to transient/irreverent abnormalities noted in 22% of patients, QTc interval prolongation to > 450 msec was noted in 2 cases [4]. In the DASISON trial, 2% vs 4% of dasatinib and imatinib arms had QTc intervals between 450-500 msec, and one patient (0.4%) in each group had a QTc interval of > 500 msec. Median changes in QTc interval from baseline were 3 msec in the dasatinib group and 8 msec in the imatinib group [12].

## Bleeding

Bleeding was noted in studies of dasatinib in the second-line setting, mostly in patients with severe thrombocytopenia and more commonly in patients with advanced disease [38]. In vitro data suggest that dasatinib reversibly inhibits platelet activation [39]. In the DASISION trial, GI bleeding or other bleeding events occurred at a similar frequency in both treatment arms (5%). One patient in the dasatinib group and two patients in the imatinib group reported a grade 3-4 bleeding event [12].

## Other nonhematologic AEs

Mild to moderate nonhematologic AEs such as headache, fatigue, muscle pains/cramps, and joint pain are commonly seen with BCR-ABL inhibitor treatment. These effects are usually easily managed without dose reduction and rarely cause dose interruptions. Recent data suggest that some of these AEs occur at different rates with dasatinib or nilotinib compared with imatinib. In the DASISION study, musculoskeletal AEs were less common with dasatinib compared with the imatinib arm, including myalgia (6% vs 12%), muscle inflammation (4% vs 17%), and musculoskeletal pain (11% v 14%). Rates of fatigue (8% v 10%) and headache (12% vs 10%) were similar in both arms. With each of these AEs, ≤ 1% of patients had a grade 3-4 event [12]. In the MDACC study of dasatinib, pain in joint/muscle (combined grouping; 74%), fatigue (73%), and headache (56%) were reported at high rates (grade 3-4 in 6%, 6%, and 2%, respectively) [13]. In the ENESTnd trial, muscle spasm occurred at a lower frequency in the nilotinib arms (6-7%) compared with the imatinib arm (24%). Myalgia occurred at a similar rate across all three arms (10%), as did fatigue (8-11%). However, headache occurred at a higher frequency in the nilotinib 300 mg BID (14%) and 400 mg BID (21%) treatment groups than in the imatinib treatment group (8%). Rates of grade 3-4 events with these AEs were ≤ 1% [14]. Similar to the MDACC study of dasatinib, the study of nilotinib at the same institution reported substantially higher rates of fatigue (67%; grade 3-4 in 3%) and headache (39%; no grade 3-4) than in the randomized study. Musculoskeletal AEs were reported as separate categories; 10% of patients experienced muscle cramp (0% grade 3-4) and 10% experienced joint pain (3% grade 3-4) [15]. In the GIMEMA study, 41% of patients taking nilotinib experienced bone/muscle/joint pain (combined grouping), of which 4% were grade 3. In addition, 30% experienced headache and 22% experienced fatigue (no grade 3-4 in each case) [4].

## Biochemical abnormalities

Rates of biochemical abnormalities vary in patients receiving different BCR-ABL inhibitors and seem to be most common during nilotinib treatment. In the DASISION trial, grade 3-4 hypophosphatemia occurred in 4% of patients treated with dasatinib compared with 21% of the patients treated with imatinib. Rates of other grade 3-4 biochemical abnormalities were low in both treatment arms, including markers of hepatic toxicity (elevated alanine aminotransferase [ALT] or aspartate aminotransferase [AST] each < 1% vs 1%, elevated total bilirubin 1% vs 0%) and pancreatic toxicity (no grade 3/4 elevations in lipase or amylase, or cases of hyperglycemia were recorded). Rates of all-grade biochemical abnormalities were not reported [40]. Four imatinib-treated patients but no dasatinib-treated patients discontinued therapy because of biochemical abnormalities [12]. In the MDACC study of dasatinib, hypophosphatemia occurred in 6% (2% grade 3-4) of patients, hyperglycemia occurred in 24% (2% grade 3-4), and elevated ALT or AST occurred in 16% and 15%, respectively (no grade 3-4 cases) [13].

In the ENESTnd trial, more nilotinib-treated patients than imatinib-treated patients had biochemical abnormalities associated with liver and pancreatic toxicity. With nilotinib 300 mg BID or 400 mg BID or imatinib, ALT was elevated in 66% vs 73% vs 20% of patients, respectively (grade 3-4 in 4% vs 9% vs 2%), AST was elevated in 40% vs 48% vs 23% (grade 3-4 in 1% vs 3% vs 1%), and bilirubin was elevated in 53% vs 62% vs 10%, (grade 3-4 in 4% vs 8% vs < 1%). Elevated lipase was observed in 24-29% of patients receiving nilotinib (6% grade 3-4) compared with 11% of patients receiving imatinib (3% grade 3-4). Respective rates of hyperglycemia were 36-41% (4-6% grade 3-4) vs 20% (no grade 3-4) and elevated amylase occurred in 15-18% vs 12% (grade 3-4 in < 1-1%) of patients. Hypophosphatemia occurred in 32-34% of nilotinib arms (5% grade 3-4) and 45% of the imatinib arm (8% grade 3-4). All newly occurring grade 3-4 biochemical abnormalities occurred within the first 2 months of therapy. Discontinuations due to biochemical abnormalities occurred in 2% of both nilotinib arms and 1% of the imatinib arm [14]. In other studies of nilotinib as front-line therapy, ALT elevation occurred in 42-48% (0-8% grade 3) of patients, AST elevation occurred in 29-46% (0-3% grade 3), and bilirubin elevation occurred in 39-53% (grade 3-4 in 7-16%) [4,15]. Elevated markers of pancreatic toxicity were reported in both studies. However, hyperglycemia was more common in the MDACC study (44%, grade 3-4 in 5%) than elevated lipase (10%, grade 3/4 in 5%) or amylase (3%, grade 3/4 in 2%), whereas hyperglycemia (12%, grade 3 in 3%) was less common in the GIMEMA study than elevated lipase (29%, grade 3-4 in 8%) or amylase (18%, grade 3 in 4%) [4,15]. One patient in the GIMEMA study discontinued treatment following lipase elevation.

Bilirubin elevation on nilotinib may be due in part to nilotinib inhibition of UGT1A1 activity. UGT1A1 catalyzes the conjugation of hepatic bilirubin and polymorphisms in the promoter region of UGT1A1 are associated with Gilbert's Syndrome (inherited mild, chronic, unconjugated hyperbilirubinemia in the absence of liver disease or overt hemolysis). Reduced UGT1A1 expression due to polymorphisms is associated with elevation of bilirubin in plasma [41,42]. *UGT1A1 *promoter polymorphism has been found to increase the risk of nilotinib-induced bilirubin elevation [43].

## Dose adjustments and discontinuations due to toxicity

The rate of discontinuations because of drug toxicity provides a measure of the frequency of the most problematic AEs. In the DASISION trial, discontinuations following study drug toxicity occurred in 5.0% of the dasatinib arm and 4.3% of the imatinib arm. Of these, hematologic toxicity led to discontinuation in 1.6% vs 1.2%, and nonhematologic toxicity led to discontinuation in 3.5% vs 3.1%, respectively. Median doses of drug delivered were 99 mg/d in the dasatinib 100 mg QD arm vs 400 mg/d in the imatinib 400 mg QD arm. Data for dose interruptions and reductions have not been reported [12]. In the ENESTnd trial, discontinuations due to AEs occurred in 5% with nilotinib 300 mg BID, 9% with nilotinib 400 mg BID, and 7% with imatinib. Median doses of drug delivered were 592 mg/d in the nilotinib 300 mg BID arm, 779 mg/d in the nilotinib 400 mg BID arm, and 400 mg in the imatinib 400 mg QD arm. Respective rates of dose reduction/interruption were 59%, 66%, and 52%. Median cumulative durations of interruptions due to AEs or biochemical abnormalities were 19 days, 22 days, and 15 days, respectively [14].

## Future directions with BCR-ABL inhibitors

### Bosutinib

Data are awaited from the randomized phase 3 trial of bosutinib vs imatinib for first-line treatment for newly diagnosed CML [37]. However, data have been reported for the efficacy and safety of bosutinib in patients with CP-CML who had prior imatinib treatment. Response rates with bosutinib were comparable to those seen in trials of dasatinib and nilotinib in the second-line setting, including CCyR in 50% and MMR in 52% of evaluated patients, of which 32% were complete. At 24 months, rates of progression-free and overall survival were 80% and 95%, respectively. Responses were similar in patients with or without BCR-ABL mutations. Safety data indicate that bosutinib has a distinct safety profile compared with currently approved BCR-ABL inhibitors. AE rates should be interpreted with caution based on previous observations with dasatinib and nilotinib that AEs generally occur more frequently with second-line (post-imatinib) treatment compared with first-line treatment. Grade 3-4 thrombocytopenia, neutropenia, and anemia occurred in 24%, 16%, and 12%, respectively of patients receiving bosutinib. GI AEs were common with bosutinib treatment, including diarrhea in 84% of patients (9% grade 3-4), nausea in 44% (2% grade 3-4), and vomiting in 36% (3% grade 3-4). In addition, 34% of patients suffered from rash (9% grade 3-4), 21% had abdominal pain (1% grade 3-4), 21% had fatigue (1% grade 3-4), 14% had headache (no grade 3-4), and 13% had joint pain (< 1% grade 3-4). Rates of fluid retention AEs were not reported, indicating a frequency of < 10%. Of grade 3-4 biochemical abnormalities, elevated ALT occurred in 10% of patients, elevated AST in 5%, elevated lipase in 7%, elevated glucose in 3%, decreased phosphate in 8%, and hypermagnesemia in 12%. In addition, 19% of patients receiving bosutinib in this study discontinued treatment due to AEs and 45% had a dose reduction due to AEs. The median dose of bosutinib was 454 mg/d (starting dose was 500 mg/d) [44]. Overall, preliminary data from this phase 1/2 trial indicate that bosutinib is an active agent for patients with CP-CML who have failed on prior imatinib treatment, with activity against a range of BCR-ABL mutations, and an acceptable toxicity profile.

## Inhibitors for T315I mutant

Resistance to imatinib or relapse in patients with CML arises most frequently because of point mutations within the BCR-ABL coding sequence [45-48]. In vitro data has shown that dasatinib, nilotinib, and bosutinib effectively inhibit the majority of mutated forms of BCR-ABL that have been associated with imatinib resistance in the clinic [6,9,49]. However, the T315I point mutation confers resistance to imatinib, dasatinib, nilotinib, and bosutinib [50,51]. Although data are not yet available to indicate how frequently T315I will cause resistance to the newer agents, this mutation represents an "Achilles' heel" for CML therapy.

Several TKIs that are active against the T315I-mutated form of BCR-ABL are being developed. MK-0457, a potent inhibitor of BCR-ABL and aurora kinases, was the first agent to show clinical activity against the T315I mutation; however, development of this drug was halted due to cardiac toxicity [52]. Other BCR-ABL/aurora kinases inhibitors with activity against T315I are in clinical development, including XL228, PHA-739358 (danusertib), and AT9283 [53-57]. Ponatinib (AP24534) is a multitargeted BCR-ABL/SRC kinase inhibitor with potent in vitro activity against all tested mutants of BCR-ABL including T315I, and clinical activity has been reported in patients with a T315I mutation [58-60]. Further clinical studies of ponatinib are ongoing, most notably a single-arm phase 2 study in patients with CML or Ph+ acute lymphoblastic leukemia (ALL) who either are resistant or intolerant to either dasatinib or nilotinib, or who harbor the T315I mutation (Ponatinib Ph+ ALL and CML evaluation [PACE]; NCT01207440). Switch pocket kinase inhibitors, such as DCC-2036 and DCC-2157, target the sites involved in controlling the conformation of BCR-ABL, which ultimately controls the activity state of the kinase. These agents are active against cells expressing a variety of BCR-ABL mutations, including T135I. A phase 1 study of DCC-2036 in patients with T315I or failure on two different TKIs is underway (NCT00827138) [61,62]. Omacetaxine (previously homoharringtonine) is a naturally occurring alkaloid derived from evergreen trees that induces apoptosis in leukemic cells, including those harbouring the T315I mutation [63-65]. In a phase 2/3 trial in patients with CML and a T315I mutation, omacetaxine treatment in the subset of patients with CP-CML resulted in a CCyR in 10% and a MMR in 15% [66]. The underlying mechanism for omacetaxine inhibitory effects on leukemic cells is still unknown. Studies of omacetaxine in patients with CML, either alone or in combination with other treatments, are ongoing.

## Authors' contributions

GW, SR and DL involved in concept design, coordination, drafting and critically revising the manuscript.
